# Supplementary material for: HCV core antigen is an alternative marker to HCV RNA for evaluating active HCV infection: implications for improved diagnostic option in an era of affordable DAAs
Source: PeerJ. 2017 Nov 6;5:e4008. doi: 10.7717/peerj.4008 (PMC5678506; doi:10.7717/peerj.4008)
Supplement: Table S1 [file peerj-05-4008-s001.docx]

**TABLE S1.** Comparison of the anti-HCV, HCV Ag and HCV RNA status in all samples.

|  | **Anti-HCV (S/CO)** | | **HCV Ag (fmol/L)** | | **Combined Anti-HCV (S/CO) and HCV Ag (fmol/L)** | | **Total (%)^b^** |
| --- | --- | --- | --- | --- | --- | --- | --- |
| **HCV RNA status** | **≥ 5.0 (%)^a^** | **< 5.0 (%)^a^** | **≥ 3 (%)^a^** | **< 3 (%)^a^** | **≥ 5 & ≥ 3 (%)^a^** | **Others (%)^a^** |  |
| **Positive** | 222 (88.1) | 0 (0.0%) | 220 (100%) | 2 (2.9%) | 220 (100.0%) | 2 (0.9%) | 222 (76.6%) |
| **Negative** | 30 (11.9%) | 38 (100.0%) | 0 (0.0%) | 68 (97.1%) | 0 (0.0%) | 68 (97.1%) | 68 (23.4%) |
| **Total (%)^b^** | 252 (86.9%) | 38 (13.1%) | 220 (75.9%) | 70 (24.1%) | 220(75.0%) | 70 (24.1%) | 290 (100.0%) |

^a^From a total of each category.

^b^From 290 samples.
