# Supplementary material for: HCV core antigen is an alternative marker to HCV RNA for evaluating active HCV infection: implications for improved diagnostic option in an era of affordable DAAs
Source: PeerJ. 2017 Nov 6;5:e4008. doi: 10.7717/peerj.4008 (PMC5678506; doi:10.7717/peerj.4008)
Supplement: Table S2 [file peerj-05-4008-s002.docx]

|  |  | **Sensitivity (%)** | **Specificity (%)** | **Positive predictive value (%)** | **Negative predictive value (%)** | **Accuracy (%)** |
| --- | --- | --- | --- | --- | --- | --- |
| **S/CO cut-off^a^ value** | **5.0** | 222/222 (100.0) | 38/68 (55.9) | 222/252 (88.1) | 38/38 (100.0) | 260/290 (89.7) |
|  | **8.0** | 221/222 (99.5) | 50/68 (73.5) | 221/239 (92.5) | 50/51 (98.0) | 271/290 (93.4) |
|  | **9.0** | 221/222 (99.5) | 51/68 (75.0) | 221/238 (92.9) | 51/52 (98.1) | 272/290 (93.8) |
|  | **10.0** | 219/222 (98.6) | 52/68 (76.5) | 219/235 (93.2) | 52/55 (94.5) | 271/290 (93.4) |
|  | **12.0** | 207/222 (93.2) | 57/68 (83.8) | 207/218 (95.0) | 57/72 (79.2) | 264/290 (91.0) |
|  | **13.0** | 183/222 (82.4) | 63/68 (92.6) | 183/188 (97.3) | 63/102 (61.8) | 246/290 (84.8) |
|  | **15.0** | 85/222 (38.3) | 67/68 (98.5) | 85/86 (98.8) | 67/204 (32.8) | 152/290 (52.4) |

**TABLE S2.** Diagnostic validity of anti-HCV at different S/CO cut-off value.

^a^Diagnostic validity at different cut-off values were compared with the HCV RNA status.
