# Supplementary material for: HCV core antigen is an alternative marker to HCV RNA for evaluating active HCV infection: implications for improved diagnostic option in an era of affordable DAAs
Source: PeerJ. 2017 Nov 6;5:e4008. doi: 10.7717/peerj.4008 (PMC5678506; doi:10.7717/peerj.4008)
Supplement: File S1 [file peerj-05-4008-s003.docx]

**File S1. Budget calculation based on data utilized from the results in this study**

**Standard strategy**

(All samples with anti-HCV reactive will be confirmed active infection with RNA assay)

|  | **Assay** | **Sample number** | **Unit cost** | | **Total cost** | |
| --- | --- | --- | --- | --- | --- | --- |
|  |  |  | **THB** | **USD** | **THB** | **USD^b^** |
|  | Anti-HCV | 298 | 250 | 8 | 74,500 | 2,237 |
|  | HCV RNA | 290^a^ | 2,000 | 60 | 58,0000 | 1,7417 |
| **Total budget** | |  |  |  | **654,500** | **19,655** |

^a^All samples with anti-HCV S/CO ≥ 1.0 were subsequently determined presence of HCV infection by RNA testing. One USD approximately equal to 33.3 THB. ^b^One USD approximately equal to 33.3 THB (Average Selling Rates, Bank of Thailand on 25/09/2017).

**Optional strategy**

(All samples with anti-HCV reactive with S/CO ≥ 5.0 will be confirmed active infection with HCV Ag assay. Only sample with anti-HCV reactive/HCV Ag non-reactive will be confirmed by RNA assay.)

|  | **Assay** | **Sample number** | **Unit cost** | | **Total cost** | |
| --- | --- | --- | --- | --- | --- | --- |
|  |  |  | **THB** | **USD** | **THB** | **USD^b^** |
|  | Anti-HCV | 298^c^ | 250 | 8 | 74,500 | 2,237 |
|  | HCV Ag | 252^d^ | 800 | 24 | 201,600 | 6,054 |
|  | HCV RNA | 32 | 2,000 | 60 | 64,000 | 1,922 |
| **Total budget** | |  |  |  | **340,100** | **10,213** |

^b^One USD approximately equal to 33.3 THB (Average Selling Rates, Bank of Thailand on 25/09/2017). ^c^Samples with anti-HCV S/CO ≥ 5.0 were subsequently determined presence of HCV infection by HCV Ag testing. ^d^Samples with discordant results of positive HCV antibody but negative to HCV Ag.

Different cost between standard and optional strategy:

654,500 – 340,100 = 314,400 Bath (~ 9441 USD)

**Budget reduction = 48%**
